# Supplementary material for: Does Insect Aversion Lead to Increased Household Pesticide Use?
Source: Insects. 2022 Jun 18;13(6):555. doi: 10.3390/insects13060555 (PMC9224736; doi:10.3390/insects13060555)
Supplement: Supplementary file 1 [file insects-13-00555-s001.zip › insects-1742277-supplementary.pdf]

Supplementary Table S1: List of household pesticide products found in participants' homes

Category	Mode of use	Brand name	Product name	Active ingredients	Pesticide family
Insecticide (for treating crawling insects)	Aerosol	Sano	K-300	Lambda-cyhalothrin 0.03%	Pyrethroid
				Prallethrin 0.1%	Pyrethroid
				Piperonyl butoxide 1.0%	Synergist
Insecticide (for treating crawling insects and flying insects)	Aerosol	Sano	Rav Kotel K	Deltamethrin 0.02%	Pyrethroid
				S- bioallethrin 0.2%	Pyrethroid
				Piperonyl butoxide 1.0%	Synergist
Insecticide (for treating crawling insects) Slow release formula	Microcapsulated, liquid spray	Sano	K-2000	Cyphenothrin 0.3%	Pyrethroid
Insecticide (for treating flying insects)	Aerosol	Sano	K-400	Tetramethrin 0.65%	Pyrethroid
				Piperonyl butoxide 0.6%	Synergist
				MGK 264 1.0%	Synergist
Insecticide (for treating crawling insects)	Aerosol	Sano	K-500	Lambda-cyhalothrin 0.03%	Pyrethroid
				Prallethrin 0.1%	Pyrethroid
				Piperonyl butoxide 1.0%	Synergist
Insecticide (for treating crawling insects)	Liquid spray	Sano	K-333	Bifenthrin 0.096%	Pyrethroid
Insecticide (for treating crawling insects)	Aerosol	Raid	Ant killer	Imiprothrin 0.1%	Pyrethroid
				Cypermethrin 0.1%	Pyrethroid
Insecticide (for treating crawling insects)	Liquid spray	RPC	Excelent	Bifenthrin 0.096%	Pyrethroid

Insecticide (for treating crawling insects and flying insects)	Solution, for exterminators	Dor.Ky	Flyco 2	Permethrin 20.0%	Pyrethroid
				Piperonyl butoxide 10.0%	Synergist
				D-limonene 3.5%,	Botanical insecticide
				Citronella oil 3.5%,	Botanical insect repellent
Insecticide (for treating flying insects)	Aerosol	Sano	Paztox	Esbiothrin 0.12%	Pyrethroid
				Tetramethrin 0.05%	Pyrethroid
				Phenothrin 0.1%	Pyrethroid
				Piperonyl butoxide 0.15%	Synergist
				MGK 264 0.45%	Synergist
